# Moderate Alcohol Consumption Inhibits Sodium-Dependent Glutamine Co-Transport in Rat Intestinal Epithelial Cells in Vitro and Ex Vivo

**DOI:** 10.3390/nu11102516

**Published:** 2019-10-18

**Authors:** Molly Butts, Raja Singh Paulraj, Jennifer Haynes, Subha Arthur, Soudamani Singh, Uma Sundaram

**Affiliations:** Department of Clinical and Translational Sciences, Joan C. Edwards School of Medicine, Marshall University, Huntington, West Virginia, USA; butts15@marshall.edu (M.B.); haynesje@marshall.edu (J.H.); arthursu@marshall.edu (S.A.); singhs@marshall.edu (S.S.)

**Keywords:** ethanol, enterocyte nutrient absorption, sodium-dependent nutrient co-transport, B0AT1

## Abstract

Malnutrition is present in chronic alcoholics. However, how moderate alcohol consumption affects the absorption of nutrients like glutamine has not been investigated. Glutamine, an amino acid, is vital to gastrointestinal health. Glutamine is absorbed via sodium-dependent glutamine co-transport (B0AT1; *SLC6A19*) along the brush border membrane of absorptive villus cells. Rat intestinal epithelial cells (IEC-18) and sixteen-week-old Sprague Dawley rats were administered the equivalent of a 0.04% blood alcohol content of ethanol (8.64 mM; 2 g/kg) to investigate the effect of moderate alcohol on sodium-glutamine co-transport. Sodium-dependent ^3^H-glutamine uptakes were performed to measure B0AT1 activity. Inorganic phosphate was measured as a function of Na-K-ATPase activity. Protein expression was analyzed by immunohistochemical and Western blot analysis. Ethanol significantly inhibited sodium-dependent glutamine absorption and Na-K-ATPase activity in enterocytes in vitro and ex vivo. Kinetic studies suggested that the mechanism of inhibition was due to decreased maximal rate of uptake (*V_max_*) of the B0AT1 co-transporter, corresponding to decreased B0AT1 protein expression and secondary to an inhibited sodium-gradient at the cellular level in vitro and ex vivo. In all, moderate ethanol significantly inhibited glutamine absorption at the level of decreased B0AT1 expression at the brush border membrane and a reduced sodium gradient, which may contribute to malnutrition present in chronic alcoholics.

## 1. Introduction

According to the National Institute of Alcohol Abuse and Alcoholism, over 15 million adults in the United States have an alcohol use disorder. Chronic alcohol use, defined by binge drinking five or more days per month, has many well documented negative consequences including an increased risk for cancer, cardiovascular disease, liver disease and malnutrition [1,2]. This increased risk of malnutrition was demonstrated in 1984, when studies conducted by the Veterans Health Administration Cooperative Studies Program presented that patients with almost 50 percent of their total energy intake from alcohol were often malnourished despite adequate caloric intake [3].

Chronic alcoholics were considered to be malnourished due to suboptimal dietary intake. However, recent research has determined that ethanol also decreases the absorption of essential vitamins along the small intestine [4]. The absorption of over 20 nutrients have been shown to be affected by chronic alcohol consumption including proteins, carbohydrates, such as glucose [5,6,7,8], lipids [9], as well as vitamins such as vitamin B_12_ [4] and vitamin C [10]. Clearly, the effect of ethanol on nutrient absorption in chronic alcoholics has been well characterized. However, few studies have examined the effect of ethanol on the individual nutrient co-transporters that are responsible for absorbing these essential nutrients. Ethanol has been shown to affect vitamin co-transporters, including thiamine [11], folate [12], riboflavin [13] and vitamin C co-transporters, which leaves a substantial gap in knowledge surrounding ethanol’s effect on many other nutrient co-transporters along the small intestine [10].

Furthermore, in over fifty years of alcohol and nutrition research, the effect of ethanol on glutamine absorption in the small intestine has never been investigated. Glutamine is a conditionally essential amino acid that is imperative for maintaining the health of the intestinal mucosa [14]. Glutamine is the primary source of energy for enterocytes and has been shown to play an important role in maintaining the integrity of the mucosa during gastrointestinal disorders like inflammatory bowel disease [15,16]. Glutamine is absorbed through several different transporter systems in the intestine including systems N, ASCT and B0 [14]. It was previously shown that a system B0 sodium-dependent glutamine co-transporter, B0AT1 (*SLC6A19*), was located at the brush border membrane (BBM) of villus cells [17]. B0AT1 is an electrogenic transmembrane protein that functions as a secondary active co-transporter. Thus, B0AT1 moves sodium and glutamine into the cell at a 1:1 ratio along sodium’s concentration gradient [14]. This concentration gradient is provided by the basolateral Na-K-ATPase and is utilized by B0AT1. Once in the body, glutamine is used in a variety of cell metabolism processes including as a substrate in the citric acid cycle, gluconeogenesis, lipogenesis and in the urea cycle [18].

In this study, we hypothesize that ethanol affects the absorption of glutamine via B0AT1 in the BBM of intestinal villus cells. To better understand the onset of alcohol-dependent malnutrition, we focused our study on a moderate dose of ethanol, defined as the amount in two alcoholic beverages per day for men and one for women, equivalent to a blood alcohol content (BAC) of 0.04%. Thus, the aim of the present study is to investigate the effect of a moderate dose of ethanol on B0AT1 in rat intestinal epithelial cells both in vitro and ex vivo.

## 2. Materials and Methods

### 2.1. Animal Studies

Thirteen to fifteen-week-old male Sprague Dawley (SD) rats were purchased from Charles River (strain code: 001) and had equal access to normal chow and water. The rats were kept in a twelve-hour light and dark cycle. All rats were allowed to acclimate to their environment for at least one week, with procedures starting at 16 weeks old. Rats were exposed to an oral intragastric gavage with a manual restraint and 2 g/kg ethanol [19] or tap water was administered to ethanol-treated rats or control-treated rats, respectively for one, three or six hours. At the three and six-hour time points, blood for plasma separation for BAC measurement was collected via tail nick. Rats in the six-hour treatment group were administered a second dose of ethanol three hours after the first dosage to maintain the correct BAC. Animals were euthanized with excess CO_2_ and a final blood sample for plasma separation was collected via cardiac puncture. BAC was measured using an AM1 Alcohol Analyzer (Analox, Stokesley, UK) and the respective Analox kit instructions. The Marshall University IACUC approved all animal handling, treatment and euthanasia in this protocol (IACUC 1175526-8).

### 2.2. Cell Culture

The immortalized non-malignant rat intestinal epithelial cell line IEC-18 (CRL-1589 American Type Culture Collection, Manassas, VA, USA) were cultured in Dulbecco’s Modified Eagle Medium (DMEM) (high glucose 4.5 g/L, sodium bicarbonate 3.7 g/L) containing 4 mM L-glutamine, 10% vol/vol fetal bovine serum, 0.02% insulin and 0.25%-hydroxybutyric acid in a humidified atmosphere of 10% CO_2_ at 37°C. Cells were grown to 4 days post confluence as monolayers, only using cells between passages 5 and 20, until ethanol treatment. The medium was changed every two to three days. Cells were treated with 8.64 mM ethanol (200 proof, Pharmco Aaper), the equivalent of a 0.04% BAC dosage, directly into fresh medium for one, three or six hours. Control-treated cells were exposed to the same volume of sterile water in fresh medium.

#### 2.2.1. Calphostin C Treatments

IEC-18 cells were pretreated with Calphostin C (Abcam, Cambridge, MA, USA, 121263-19-2; 200 nM) for one hour before ethanol treatment. Then, IEC-18 cells were co-treated with Calphostin C and ethanol, as determined by previous studies [20].

#### 2.2.2. siRNA Transfections

Predesigned scrambled siRNA as a negative control (Thermo Fisher, Waltham, MA, USA, AM4635) and protein kinase C (PKC) targeted specific siRNA (Thermo Fisher s128272 and s128273) were pooled and used for siRNA transfections. Individual siRNAs (1.5 μg each) were suspended in a nucleofector solution (pH 7.4, 7.1 mM ATP, 11.6 mM MgCl_2_.6H_2_O, 13.6 mM NaHCO_3_, 84 mM KH_2_PO_4_ and 2.1 mM glucose). Using IEC-18 cells, both the negative control and PKC siRNA was nucleofected into IEC-18 cells using a Nucleofector II device (Lonza, Basel, Switzerland) according to the manufacturer’s instructions. Transfected cells were grown, treated and used for experiments as described above.

### 2.3. Na-K-ATPase Measurement

Control and ethanol-treated IEC-18 cells were washed twice with ice-cold phosphate-buffered saline and cells were harvested by scraping. Isolated villus cells from rats were homogenized and centrifuged at 8000× *g* for five min. Inorganic phosphate (P*_i_*) formation in cellular homogenates was measured as indirect Na-K-ATPase activity as previously described [21].

### 2.4. Villus Cell Isolation

Villus cells were isolated from control and ethanol-treated rats from the terminal small intestine by a previously described Ca^+2^ chelation technique [22]. Briefly, a 12-inch section of the distal small intestine was filled with a buffer containing 0.15 mM EDTA, 112 mM NaCl, 25 mM NaHCO_3_, 2.4 mM K_2_PO_4_, 0.4 mM KH_2_PO_4_, 2.5 mM L-glutamine, 0.5 mM β-hydroxybutyrate and 0.5 mM dithiothreitol and gassed with 95% O_2_ and 5% CO_2_, pH 7.4 at 37°C. The intestine was incubated for three minutes and palpitated for three minutes to facilitate cell dispersion. The resulting cell suspension was then used for whole cell uptakes or phenyl-methyl sulfonyl fluoride was added and centrifuged at 100× *g* for three minutes and the cellular pellet was frozen immediately in liquid nitrogen and stored at −80°C for future use.

### 2.5. Protein Quantification

Using the Bio-Rad DC protein assay kit (Hercules, CA, USA), total protein was measured by the Bradford method. Bovine serum albumin (BSA) was used as a standard.

### 2.6. Protein Expression Studies

#### 2.6.1. Western Blot Studies

Cells for whole cell homogenate preparation were treated, collected and centrifuged at 8000× *g* at 4°C for five min. The resulting pellet was solubilized in RIPA buffer containing protease inhibitors (Santa Cruz Biotechnology Inc., Dallas, TX, USA). The cellular extracts in RIPA were rocked for at least one hour at 4 °C and then centrifuged at 8000× *g* at 4°C for five minutes. The resulting supernatant was measured for protein content using a NanoDrop Spectrophotometer (Thermo Scientific, Waltham, MA, USA). For the BBM protein preparation, the BBM protein was collected, solubilized and measured as described previously [17,23]. Extracted proteins were separated on an 8% polyacrylamide gel and transferred onto a polyvinylidene fluoride membrane. Membranes were blocked in 5% BSA for one hour as previously described [15,16]. Then, the membranes were incubated with rabbit monoclonal primary antibodies against B0AT1 (Abcam 180516) at 1:1000 dilution at 4°C overnight. Membranes were washed and incubated with horseradish peroxidase-conjugated anti-rabbit secondary antibodies at 1:1000 dilution at room temperature for one hour. For IEC-18 cells, the proteins were separated on an 12% polyacrylamide gel and the primary antibodies were diluted to 1:500. Otherwise, the conditions remained the same. ECL Western Blotting Detecting Reagent was used to detect the immobilized proteins. Luminescence was detected and analyzed by densitometry using a FluorChem M imager (Alpha Innotech, San Leandro, CA, USA). All blots were stripped and re-probed with mouse monoclonal primary antibodies against ezrin (MilliporeSigma, Burlington, MA, USA, MAB3822-C) at 1:1000 dilution as above and proteins were normalized against the ezrin values.

#### 2.6.2. Immunohistochemistry (IHC)

A half inch of terminal small intestine was separated for IHC. The intestine was gently washed with room temperature phosphate buffered saline (PBS), incubated for a minute in Tissue Plus O.C.T. Compound (Fisher HealthCare) and then flash-frozen in dry ice and 2-methylbutane and stored at −80°C until processing. Using a cryostat (Leica, Wetzlar, Germany, CM1806), samples were sectioned 5 μm thick at −20°C. Sections were fixed in −20°C methanol for 30 s. Non-specific binding of primary antibodies was blocked using 5% BSA for one hour at room temperature. Cells were then washed in room temperature PBS, 3 times for five min each and incubated with B0AT1 primary antibodies (Abcam 180516) at a 1:500 dilution for one hour. Cells were washed as before, then incubated with goat anti-rabbit Alexa Fluor 594 secondary antibodies (Invitrogen Life Technologies, Carlsbad, CA, USA) for one hour at a 1:500 dilution. The tissue sections were washed again, mounted with Fluoroshield Mounting Medium with 2′,6-diamidino-2-phenylindole (DAPI) (Abcam), imaged on an EVOS FL Auto 2 microscope (Invitrogen Life Technologies, Carlsbad, CA, USA) using a 20× objective and quantified with AlphaView software version 3.4.0.0.

#### 2.6.3. Immunocytochemistry (ICC)

Control and ethanol-treated cells grown on glass coverslips were fixed with ice-cold methanol for 4 min. The cells were processed with the same concentrations of blocking and secondary antibodies as above. The B0AT1 primary antibodies (Abcam 180516) were diluted at 1:250. The cells were washed, mounted, imaged using a 20× objective and analyzed as above.

### 2.7. Uptake Studies

As previously described [24], sodium-dependent uptakes were conducted using Na-HEPES buffer (47 mM KCl, 1 mM MgSO_4_, 1.2 mM KH_2_PO_4_, 20 mM HEPES, 125 mM CaCl_2_ and 130 mM NaCl; pH 7.4). Uptakes were performed with sodium using 130 mM NaCl-HEPES and without sodium using a buffer containing 130 mM TMA-Cl, the Na-free HEPES buffer. By including the sodium-free condition, the passive diffusion of glutamine can be measured. Both reaction buffers contained 200 μM of cold *L*-glutamine and 10 μCi ^3^H-*L*-glutamine. Uptake studies in IEC-18 cells were performed for exactly two minutes and stopped with ice-cold TMA-HEPES. The cells were dissolved in 1 M NaOH for 20 min at 80°C and then placed in 4 mL scintillation fluid and kept overnight. Samples were measured in a scintillation counter (LS 6500; Beckman Coulter, Fullerton, CA, USA).

Uptake studies of 100 mg of intact whole villus cells (wet weight) were conducted immediately following the villus cell isolation. Cells were washed and suspended in the 37°C Na-HEPES buffer described above. In room temperature vials, 100 μL of the previously described reaction mixtures were added to 100 μL of the cellular suspension. The uptake was halted at exactly two minutes with ice-cold stop solution. The mixture was filtered on 0.65 μm mixed cellulose esters membrane filters (DAWP; Millipore) and washed twice with ice-cold stop solution using vacuum filtration. Each filter was dissolved in 4 mL scintillation solution and radioactivity was measured as above.

#### 2.7.1. Brush Border Membrane Vesicle (BBMV) Preparation

Brush border membrane vesicles (BBMVs) from rat intestinal villus cells were prepared as previously described [17,23]. In brief, by CaCl_2_ precipitation and differential centrifugation, BBMVs were formed and incubated for an hour in a sodium-free buffer at room temperature and 5 μL of BBMVs were incubated in 95 μL reaction buffer as described above. The reaction was halted at 90 s with ice-cold stop solution, filtered on a 0.45 μm mixed cellulose esters membrane filters (HAWP; Millipore), washed twice with ice-cold stop solution using vacuum filtration and then dissolved in scintillation fluid and measured using a scintillation counter as previously stated.

#### 2.7.2. Kinetics

In IEC-18 cells, sodium-dependent glutamine uptakes were performed at 30 s at varying concentrations of glutamine (0.1, 0.5, 1, 5, 10, 25, 75 and 100 mM) as described above. Additionally, isolated rat villus cells were used to create BBMVs and kinetics were performed at six seconds at varying concentrations of glutamine (0.2, 0.5, 1, 5, 10, 25, 50, 75 and 100 mM). Uptake values were evaluated by Michaelis-Menten kinetics using a non-linear regression data analysis using Prism 7 software (GraphPad, San Diego, CA, USA).

### 2.8. Statistical Analysis

Each number (n) for any set of experiments refers to cell preparations from different passages or individual animals. Data were computed in triplicate and means ± standard error were evaluated. P-values were derived by unpaired t-tests or two-way ANOVA using Prism 7 software (GraphPad, San Diego, CA, USA). Experiments using more than one timepoint had their p-value adjusted for multiple hypotheses using a Bonferroni correction. A *p*-value of less than 0.05 was considered statistically significant.

## 3. Results

### 3.1. The Effect of Moderate Ethanol on Body Weight and BAC in SD Rats Administered Via Oral Gavage

**Figure A1:**
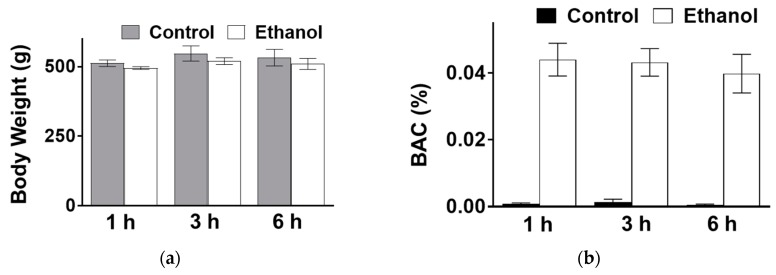
Establishment of the intragastric gavage model in Sprague Dawley (SD) rats using a moderate dosage of ethanol. (**a**) The body weights of 16-week-old SD rats did not differ between treatment groups (*p* > 0.05, *n* = 4). (**b**) Rats were administered a 2 g/kg dosage of ethanol or water. The BAC of the rats administered ethanol reached 0.04%. P-values were adjusted for multiple hypotheses using a Bonferroni correction (* 1 h: *p* < 0.01; 3 h: *p* < 0.01, 6 h: *p* < 0.05; *n* = 4). The error bars represent the standard error of the mean (SEM).

To investigate the effect of moderate ethanol on the gastrointestinal absorption of glutamine, Sprague Dawley rats were administered a moderate dosage of ethanol by intragastric gavage. The body weights of the rats did not differ (Appendix B, Figure A1a) and the appropriate BAC of 0.04% was achieved (Appendix B, Figure A1b).

#### 3.1.1. The Effect of Moderate Ethanol on Sodium-Dependent Glutamine Uptake in Ex Vivo Intestinal Epithelial Cells

**Figure 1:**
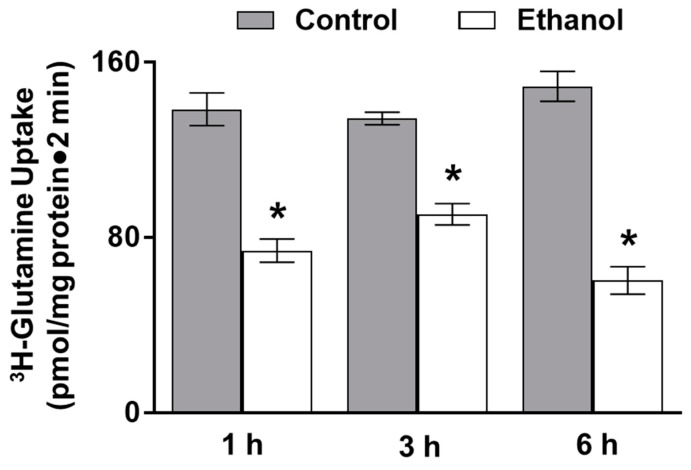
Moderate ethanol significantly inhibited glutamine absorption in SD rats. Sodium-dependent glutamine activity was determined using ^3^H-glutamine uptakes with and without a sodium-free condition in control and ethanol-treated rats (* *p* < 0.01, *n* = 4). The error bars represent the SEM. P-values were adjusted for multiple hypotheses using a Bonferroni correction (1 h: *p* < 0.01; 3 h: *p* < 0.01; 6 h: *p* < 0.01).

In isolated intestinal villus cells, ^3^H-glutamine uptakes were conducted to examine the effect of moderate alcohol consumption on glutamine absorption. Sodium-dependent glutamine absorption was significantly inhibited at all time points in the ethanol-treated rats (Figure 1; *p* < 0.01, *n* = 4).

#### 3.1.2. The Effect of Moderate Ethanol on Ex Vivo Na-K-ATPase Activity

**Figure 2:**
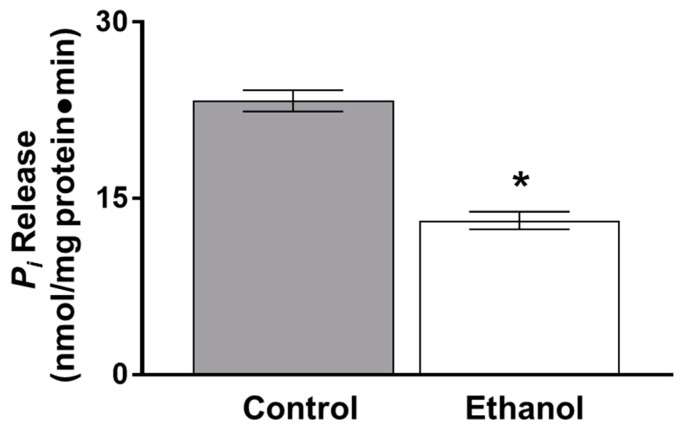
Moderate ethanol significantly inhibited Na-K-ATPase activity in Sprague Dawley (SD) rats. Na-K-ATPase activity was measured as a function of inorganic phosphate (P_i_) release following one hour of ethanol exposure (* *p* < 0.01, *n* = 4). The error bars represent the SEM.

As there was a significant inhibition of glutamine absorption following one hour of ethanol exposure in SD rats, the following experiments were conducted at the one-hour time point. Since the Na-K-ATPase establishes the concentration gradient that sodium-dependent glutamine co-transport relies on, Na-K-ATPase activity was measured ex vivo. Na-K-ATPase activity was also significantly inhibited in ethanol-treated rats at one hour (Figure 2; *p* < 0.01, *n* = 4).

#### 3.1.3. The Effect of Ethanol on Sodium-dependent Glutamine BBMV Uptakes in SD Rats

**Figure 3:**
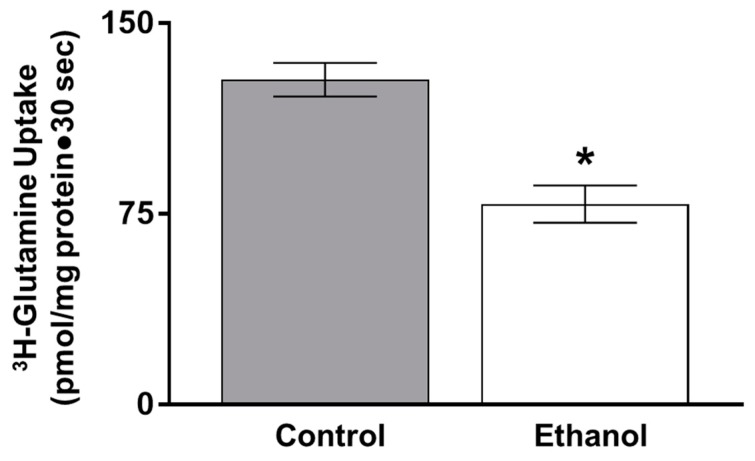
Moderate ethanol significantly inhibited ^3^H-glutamine uptake in brush border membrane vesicles (BBMVs) from intestinal villus cells of SD rats. Sodium-dependent glutamine activity was determined using ^3^H-glutamine uptakes with and without a sodium-free condition in BBMVs isolated from control and ethanol-treated rats for one hour (* *p* < 0.01, *n* = 4). The error bars represent the SEM.

Ethanol-mediated inhibition of glutamine absorption may occur independently of the Na-K-ATPase. Therefore, BBMVs were formed which separate the BBM from the basolateral membrane (BLM), where the Na-K-ATPase is localized, to remove the influence of the Na-K-ATPase. ^3^H-glutamine uptakes conducted in isolated BBMVs from SD rats treated with moderate ethanol for one hour showed that sodium-dependent glutamine absorption was significantly inhibited (Figure 3; *p* < 0.01, *n* = 4).

#### 3.1.4. The Effect of Moderate Ethanol on the Kinetic Parameters of Sodium-Dependent Glutamine Co-Transport in SD Rats

Kinetic studies showed the mechanism of inhibition of sodium-dependent glutamine co-transporter ex vivo was secondary to a decrease in the maximal rate of uptake of the co-transporter (*V_max_*; Table 1), suggesting decreased co-transporter number. There was no significant change in the affinity of the co-transporter to glutamine (1/*K_m_*).

#### 3.1.5. Protein Expression of B0AT1 in SD Rats Exposed to Moderate Ethanol

**Figure 4:**
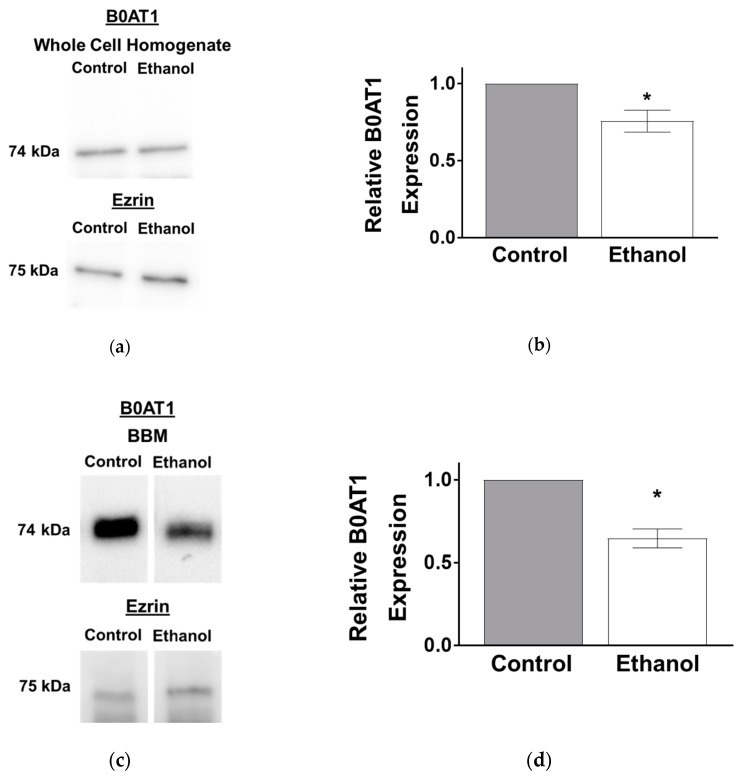
Moderate ethanol decreased the protein expression of B0AT1 in SD rats. (**a**) Western blot analysis of B0AT1 protein expression in whole cell homogenates from control and ethanol-treated rats. The blots show a representative sample used for densitometric quantification. (**b**) Densitometric quantification of B0AT1 protein expression was significantly decreased in whole cell homogenates after one-hour exposure of ethanol (* *p* < 0.01, *n* = 4). (**c**) Western blot analysis of BBM B0AT1 protein expression from control and ethanol-treated rats. The blots show a representative sample used for densitometric quantification. Each lane was located on the same membrane. (**d**) Densitometric analysis of B0AT1 protein expression was significantly decreased in the BBM fraction after one-hour exposure of ethanol (* *p* < 0.01, *n* = 4). The error bars represent the SEM. To assure equivalence of loading, the blots were normalized with anti-ezrin antibodies.

The kinetic studies displayed a significant decrease in the maximal rate of uptake for B0AT1, which suggests a decrease in co-transporter number. In order to support this finding, the protein expression of B0AT1 was examined using Western blot analysis. B0AT1 protein levels in whole cell homogenates were significantly decreased in SD rats after one-hour exposure to ethanol, as quantified by densitometry (Figure 4a,b; *p* < 0.01, *n* = 4).

B0AT1 is located in the BBM of intestinal villus cells. Therefore, the protein expression of B0AT1 was investigated at the BBM as well. B0AT1 protein expression levels were significantly decreased in the BBM of intestinal villus cells from SD rats after one-hour exposure of moderate ethanol, as quantified by densitometry (Figure 4c,d; *p* < 0.01, *n* = 4).

**Figure 5:**
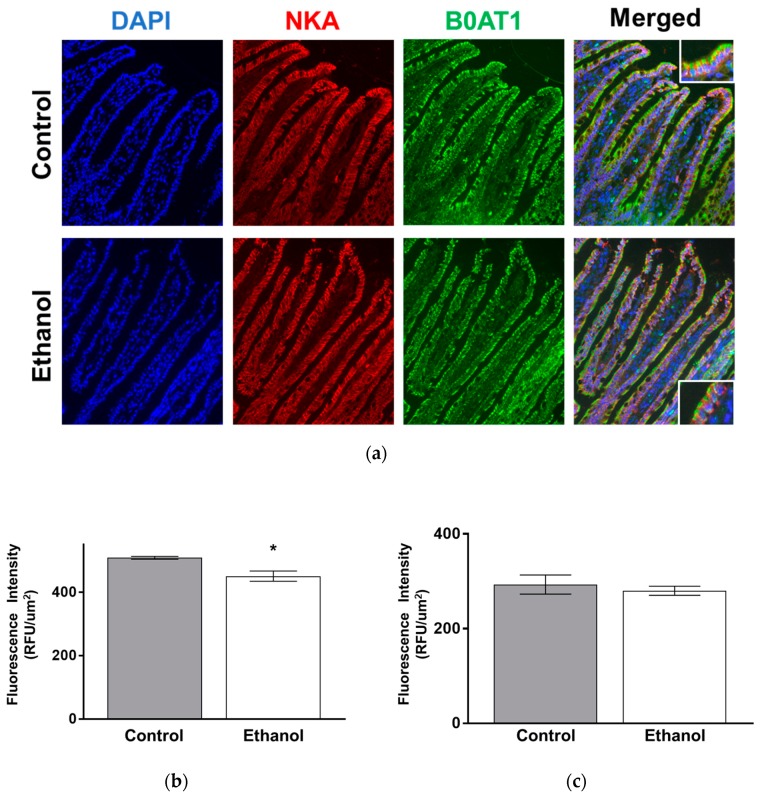
One hour of moderate ethanol significantly decreased the immunofluorescence intensity of B0AT1 in SD rats. (**a**) Shown are representative images taken using a 20× objective. The green stain represents B0AT1. The red stain represents the Na-K-ATPase α-1 subunit (NKA). The blue stain represents DAPI. (**b**) Quantification of immunofluorescence intensity of B0AT1 in SD rats (* *p* < 0.05, *n* = 4). (**c**) Quantification of immunofluorescence intensity of the Na-K-ATPase α-1 subunit (*p* > 0.05, *n* = 4). Each sample is an average of five different images obtained using a 20× objective. The error bars represent the SEM.

**Figure A2:**
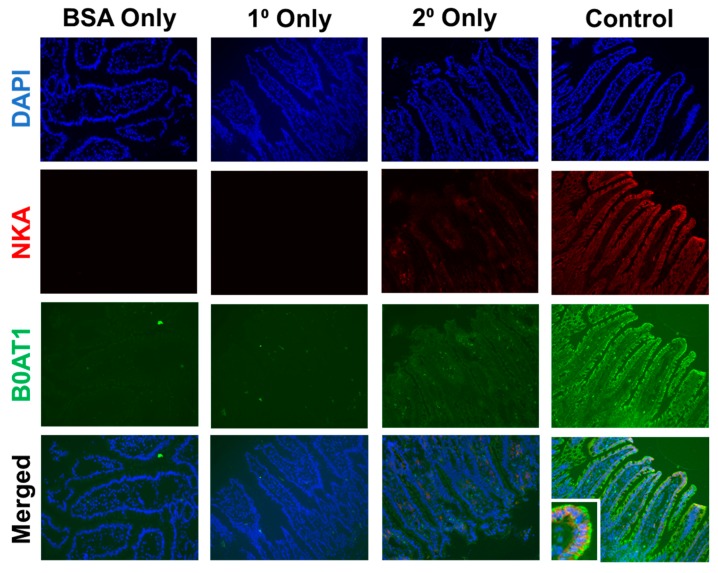
Negative controls for the B0AT1 antibodies showed no significant non-specific binding. Shown are representative images taken using a 20× objective. The green stain represents B0AT1. The red stain represents the Na-K-ATPase α-1 (NKA) subunit. The blue stain represents DAPI.

In order to better visualize this phenomenon in the intact intestinal tissue, immunohistochemical staining was conducted (Appendix B, Figure A2). Quantification of immunofluorescence intensity of B0AT1 showed that ethanol-treated rats had significantly less B0AT1 protein expression along the BBM of villus cells compared to control rats (Figure 5a,b; *p* < 0.05, *n* = 4). Moreover, the immunofluorescence intensity of the Na-K-ATPase did not change between control and ethanol-treated rats (Figure 5c; *p* > 0.05, *n* = 4).

Altogether, the molecular and kinetic data clearly show that moderate ethanol, at a dosage equivalent to a BAC of 0.04%, significantly inhibited the sodium-dependent glutamine co-transporter B0AT1 ex vivo secondary to both a decrease in the number of co-transporters at the BBM and a decreased sodium gradient.

### 3.2. The Effect of Moderate Ethanol on Sodium-Dependent Glutamine Uptake in IEC-18 Cells

**Figure A3:**
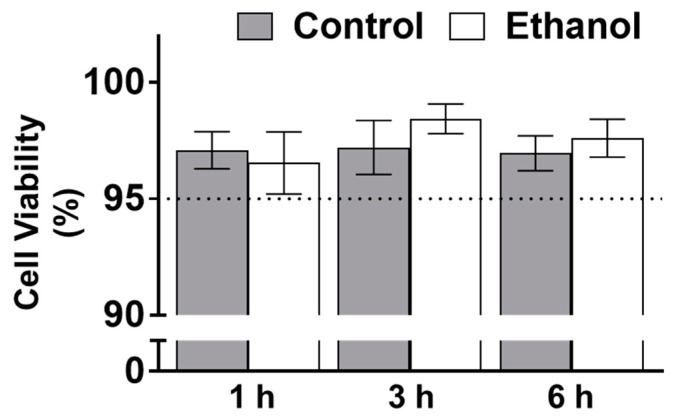
Moderate ethanol does not significantly decrease cell viability in IEC-18 cells using trypan blue exclusion (*n* = 4). Cell survival between 95–100% were considered within normal physiological parameters. Error bars represent the SEM and p-values were adjusted for multiple hypotheses using a Bonferroni correction (1 h: *p* = 2.21, 3 h: 1.16, 6 h: 1.73).

To determine if moderate ethanol affects glutamine absorption at a cellular level, cell viability was measured in IEC-18 cells exposed to moderate ethanol (Appendix B, Figure A3). There was no significant decrease in the viability of IEC-18 cells exposed to moderate ethanol between one and six hours of treatment (*p* > 0.05, *n* = 4).

**Figure 6:**
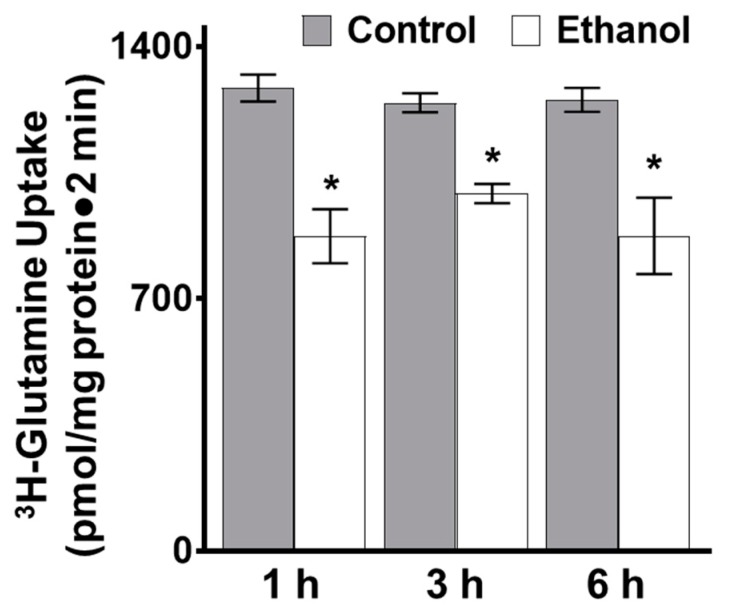
Moderate ethanol significantly inhibited glutamine absorption in IEC-18 cells. Sodium-dependent glutamine activity was determined using ^3^H-glutamine uptakes with and without a sodium-free condition in the presence and absence of moderate ethanol (* *p* < 0.05, *n* = 6). The error bars represent the SEM. P-values were adjusted for multiple hypotheses using a Bonferroni correction (1 h: *p* < 0.01; 3 h: *p* < 0.05; 6 h: *p* < 0.01).

Next, in vitro ^3^H-glutamine uptakes were conducted in IEC-18 cells to determine if moderate ethanol affects glutamine absorption. Sodium-dependent glutamine uptake was inhibited in IEC-18 cells at the one, three and six-hour timepoints, consistent with results obtained ex vivo (Figure 6; *p* < 0.01, *n* = 6).

#### 3.2.1. The Effect of Moderate Ethanol on Na-K-ATPase Activity in IEC-18 Cells

**Figure 7:**
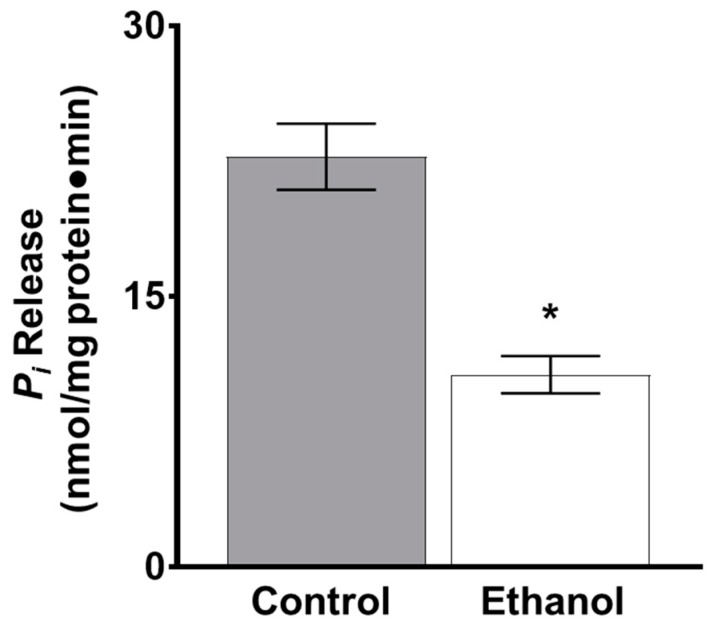
Moderate ethanol inhibited Na-K-ATPase activity in IEC-18 cells. The activity of the Na-K-ATPase was measured as a function of *P_i_* release in the presence and absence of moderate ethanol (* *p* < 0.01, *n* = 6). The error bars represent the SEM.

Na-K-ATPase activity, which maintains the transcellular sodium gradient necessary for sodium-dependent nutrient co-transporters to function properly, was significantly inhibited by ethanol in IEC-18 cells, similar to ex vivo results (Figure 7; *p* < 0.05, *n* = 6).

#### 3.2.2. The Effect of Moderate Ethanol on the Kinetic Parameters of Sodium-Dependent Glutamine Co-Transport in IEC-18 Cells

Kinetic studies showed that the mechanism of inhibition of sodium-dependent glutamine co-transport in IEC-18 cells after one-hour exposure to ethanol was secondary to a reduction in the maximal rate of uptake (*V_max_*; Table 2) of the co-transporter without a change in the affinity of the co-transporter (*K_m_*), suggesting decreased B0AT1 protein expression.

#### 3.2.3. Protein Expression of B0AT1 in IEC-18 Cells Exposed to Moderate Ethanol

**Figure 8:**
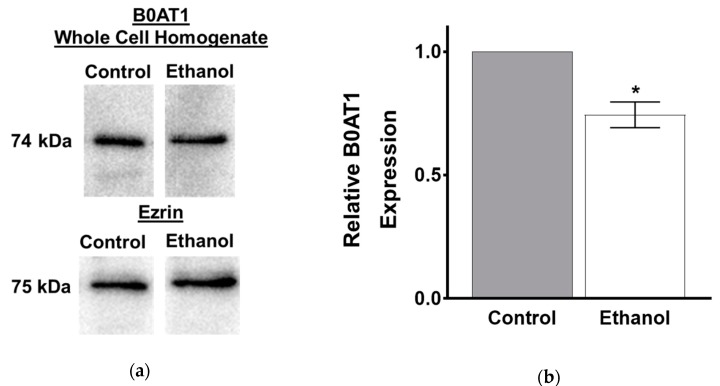

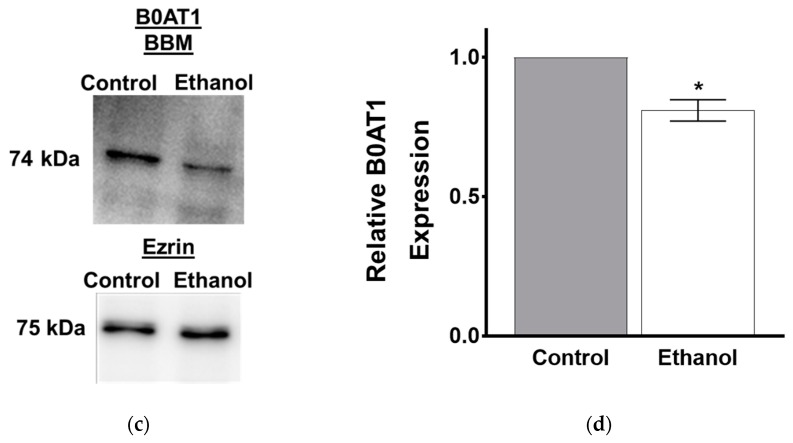
Ethanol decreased the protein expression of B0AT1 in IEC-18 cells. (**a**) Western blot analysis of B0AT1 protein expression in whole cell homogenates from control and ethanol-treated cells. The blots show a representative sample used for densitometric quantification. (**b**) Densitometric quantification of B0AT1 protein expression was significantly decreased in whole cell homogenates after one-hour exposure of ethanol (* *p* < 0.05, *n* = 6). (**c**) Western blot analysis of BBM B0AT1 protein expression from control and ethanol-treated cells. The blots show a representative sample used for densitometric quantification. (**d**) Densitometric analysis of B0AT1 protein expression was significantly decreased in the BBM fraction after one-hour exposure of ethanol (* *p* < 0.05, *n* = 4). All lanes were run on the same membrane. The error bars represent the SEM. To assure equivalence of loading, the blots were normalized with anti-ezrin antibodies.

The protein expression of B0AT1 in whole cell homogenates was significantly decreased in IEC-18 cells after one hour of ethanol treatment (Figure 8a), which was confirmed by densitometric quantitation (Figure 8b; *p* < 0.05, *n* = 6). B0AT1 protein expression levels were also decreased in the BBM of IEC-18 cells exposed to one hour of ethanol (Figure 8c), as confirmed by densitometric quantitation (Figure 8d; *p* < 0.01, *n* = 4).

**Figure 9:**
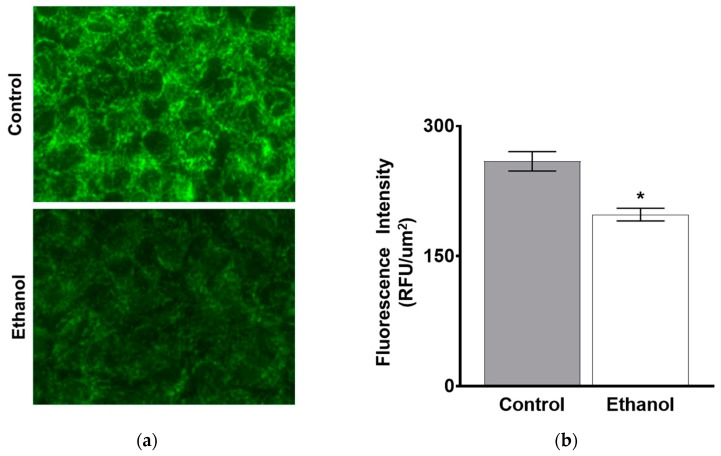
One hour of moderate ethanol significantly decreased B0AT1 immunofluorescence intensity in IEC-18 cells. (**a**) Shown are representative images taken using a 60× objective. The green stain represents B0AT1. (**b**) Quantification of B0AT1 immunofluorescence intensity. Each sample is an average of five different images obtained using a 20× objective. The error bars represent the SEM (* *p* < 0.05, *n* = 4).

In addition, immunocytochemical staining was performed in control and ethanol-treated IEC-18 cells exposed to one hour of ethanol. Consistent with the Western blot data, B0AT1 immunofluorescence intensity was significantly decreased in intestinal epithelial cells exposed to ethanol (Figure 9a,b). These data, along with the kinetic analysis above, demonstrate that the mechanism of ethanol-mediated sodium-dependent glutamine co-transport inhibition is secondary to a decrease in B0AT1 protein expression in IEC-18 cells in the BBM.

### 3.3. The Effect of Acetaldehyde on Glutamine Uptake in IEC-18 Cells

**Figure A4:**
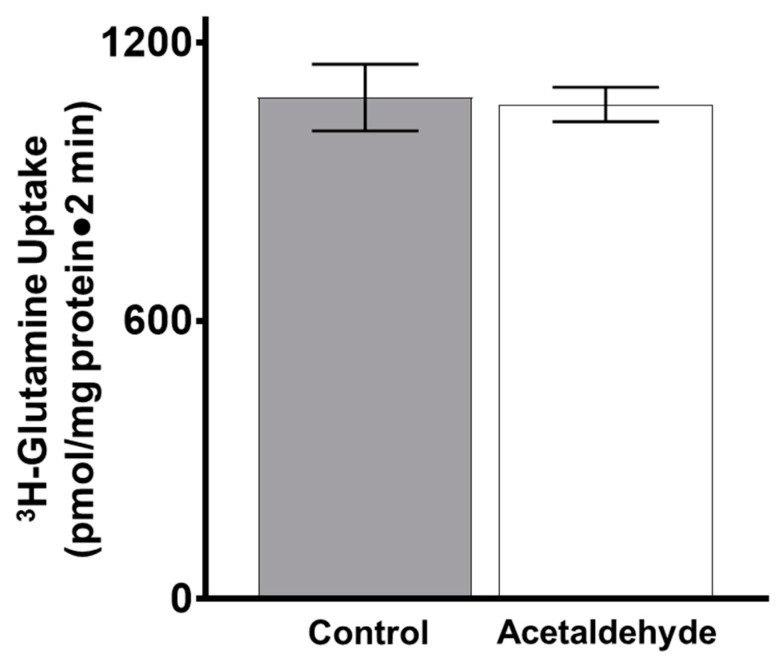
Acetaldehyde did not alter glutamine absorption in IEC-18 cells. Sodium-dependent glutamine activity was determined using ^3^H-glutamine uptakes with and without a sodium-free condition in the presence and absence of 15 μM acetaldehyde after one hour (*p* > 0.05, *n* = 4). The error bars represent the SEM.

In order to elucidate the cellular mechanism of ethanol exposure on glutamine absorption, the primary toxic metabolite of ethanol metabolism, acetaldehyde, was investigated (Appendix A). Acetaldehyde can cause cellular damage at extremely low levels [25]. Slightly more than 10 μM acetaldehyde was produced following one hour of moderate ethanol exposure (11.4 μM; data not shown). In a separate experiment, to determine if this level of acetaldehyde affects B0AT1 activity, 15 μM acetaldehyde was directly added to the cell culture medium of IEC-18 cells for one hour. Glutamine uptake did not change, suggesting that ethanol, not acetaldehyde, is the primary cause for the decrease in sodium-dependent glutamine absorption (Appendix B, Figure A4; *p* > 0.05, *n* = 4).

### 3.4. The Effect of PKC Inhibition on Glutamine Absorption in IEC-18 Cells Exposed to Ethanol

**Figure 10:**
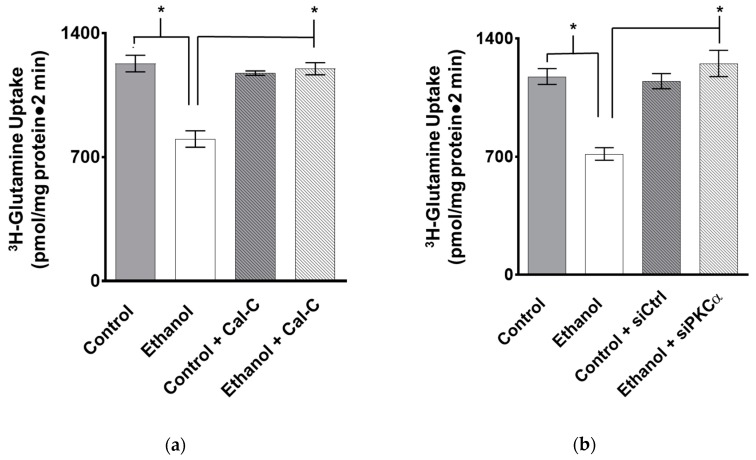
Inhibition of PKC in IEC-18 cells prevents the effect of moderate ethanol. (**a**) Exposure of the PKC inhibitor Calphostin C in IEC-18 cells reverses the ethanol-mediated inhibition of sodium-dependent glutamine absorption (* *p* < 0.01, *n* = 4). (**b**) Transfection with PKCα-siRNA (siPKCα) in IEC-18 cells prevents the ethanol-mediated inhibition of sodium-dependent glutamine absorption when compared to negative control siRNA (siCtrl; * *p* < 0.05, *n* = 3). Sodium-dependent glutamine activity was determined using ^3^H-glutamine uptakes with and without a sodium-free condition in the presence and absence of moderate ethanol. The error bars represent the SEM. P-values were tabulated using a two-way ANOVA using a Tukey’s multiple comparison test.

The protein kinase C (PKC) pathway has been shown to be involved in nutrient co-transport in IEC-18 cells [20] and following ethanol administration [26]. To elucidate a possible mechanism of action for ethanol on B0AT1, IEC-18 cells were exposed to the PKC inhibitor Calphostin C which prevented moderate ethanol’s effect on sodium-dependent glutamine co-transport (Figure 10a; *p* > 0.05, *n* = 4). Therefore, PKC is involved in the cellular mechanism of ethanol-mediated inhibition of sodium-dependent glutamine co-transport.

**Figure A5:**
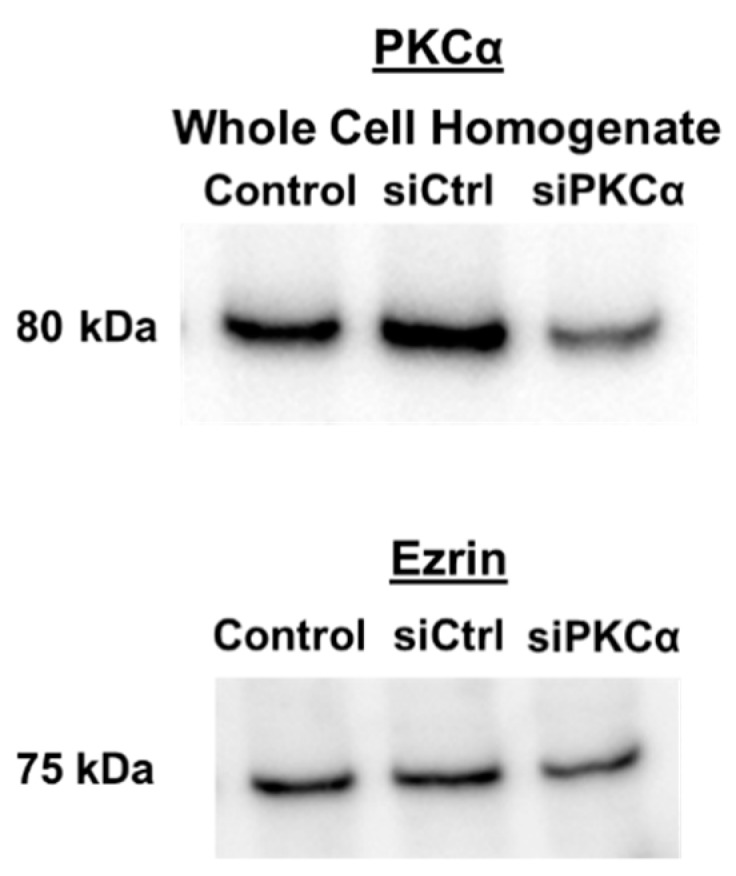
Inhibition of PKCα in IEC-18 cells. Transfection with PKCα siRNA (siPKCα) led to a reduction of the protein expression of PKCα compared to non-transfected IEC-18 cells. Negative control siRNA did not differ from non-transfected IEC-18 cells. Cells were grown from the same passage.

PKCα expression was selectively silenced in IEC-18 cells (Appendix B, Figure A5). PKCα siRNA-transfected IEC-18 cells prevented ethanol’s effect on sodium-dependent glutamine absorption (Figure 10b; *p* > 0.05, *n* = 3) which indicated that PKCα is likely important in the ethanol-mediated inhibition of B0AT1.

Overall, moderate ethanol significantly inhibits glutamine absorption via B0AT1 through an inhibition in the maximal rate of uptake of the B0AT1 co-transporter at the BBM, through decreased B0AT1 protein expression, secondary to an inhibited sodium gradient both ex vivo and in vitro. Moreover, this ethanol-mediated inhibition of B0AT1 occurs at a cellular level via a PKC-mediated pathway in intestinal epithelial cells.

## 4. Discussion

Chronic alcoholics are commonly malnourished, which is in part due to a suboptimal diet but may also be due to ethanol-mediated decreased absorption of various nutrients in the small intestine, including thiamine [27,28,29], folate [30,31], riboflavin [13], glucose [6,7,8] and minerals [4]. However, how ethanol affects the absorption of the amino acid glutamine has not yet been investigated. In this study, it was demonstrated that administration of a moderate dose of ethanol, equivalent to a 0.04% BAC, significantly decreased sodium-dependent glutamine uptake in rat intestinal epithelial cells. This is the first report of an inhibition in glutamine absorption in response to any dosage of ethanol.

In this study, we demonstrated that a moderate dose of ethanol inhibits sodium-dependent glutamine absorption in both intestinal epithelial cells of SD rats and IEC-18 cells. The mechanism by which ethanol inhibits the BBM-located sodium-dependent glutamine co-transporter B0AT1 is through a decrease in the protein expression of B0AT1. Similar to our study, Subramanya and colleagues have shown that the protein expression levels of vitamin co-transporters are decreased by ethanol [11]. Thiamine co-transport was shown to be decreased along the jejunal BBM and BLM by a reduction in the protein and mRNA expression of thiamine transporter-1 but not thiamine transporter-2, after male Wistar rats were exposed to the Lieber-DeCarli ethanol liquid diet (36% calorically ethanol) for 2, 4 and 6 weeks. This inhibition of protein and mRNA expression was linked to alterations at the promotor region but more research is needed to fully elucidate this mechanism [11]. Furthermore, in another study, the vitamin B_2_ (riboflavin) transporters 1 and 3 mRNA and protein expression were decreased when Wistar rats were fed a Lieber-DeCarli ethanol liquid diet for four weeks. The molecular mechanisms of this phenomenon have not yet been investigated [13]. The results of our present study are consistent with these previous reports and thus further the current knowledge on ethanol-dependent malnutrition.

However, it should be noted that B0AT1 is not the only sodium-dependent glutamine co-transporter along the intestine. Another sodium-dependent glutamine transporter, called ASCT2 (*SLC1A5*), may also be affected by ethanol [14]. Previous studies have shown that ASCT2 and B0AT1 can be mutually altered by leptin, which decreased the protein expression of ASCT2 and B0AT1 at the BBM of enterocytes [32]. Therefore, future studies should investigate the effect of ethanol on this and other glutamine transporters as well.

Furthermore, this study demonstrated that there was an inhibition in the Na-K-ATPase activity in villus cells from SD rats and in IEC-18 cells exposed to moderate ethanol. The Na-K-ATPase is vital in establishing the sodium gradient necessary for all sodium-dependent nutrient co-transporters to function at optimal levels. By decreasing the activity of the Na-K-ATPase, moderate ethanol may decrease BBM glutamine co-transport by affecting the sodium gradient necessary for this co-transporter to properly function. Other studies have investigated the effect of ethanol on Na-K-ATPase activity and found that acute doses of ethanol decreased the activity of the Na-K-ATPase but chronic doses of ethanol increased its activity [33,34,35]. In all, while ethanol clearly has a wide range of effects on the Na-K-ATPase, the effect of moderate ethanol on the BLM Na-K-ATPase appears to have a role in regulating sodium-dependent nutrient assimilation by enterocytes ex vivo and in vitro.

The published studies described above were investigated using in vivo models. However, in addition to using an ex vivo model, we were able to present our results in a rat intestinal epithelial cell line. By studying this novel phenomenon in a cellular model system, the mechanism was able to be further investigated. First, it was important to investigate the primary toxic metabolite of ethanol, acetaldehyde, which has been shown to increase cellular junction permeability [25], redistribute cellular junction proteins [36,37] and form toxic adducts in intestinal epithelial cells [38]. However, acetaldehyde administration did not alter glutamine absorption, suggesting that the root mechanism of action of moderate ethanol on glutamine absorption in intestinal epithelial cells remained elsewhere.

With further mechanistic studies, we demonstrated that inhibiting PKC leads to a reversal in ethanol’s inhibitory effect on glutamine absorption in IEC-18 cells. PKC has been shown to be linked to ethanol and intestinal epithelial cell co-transport functions in the literature. Our laboratory has previously demonstrated that PKCα can regulate the sodium-alanine co-transporter ASCT1 in intestinal epithelial cells [20]. In response to ethanol, PKC has also been shown to alter the function of the Na-K-ATPase [39] and ion channels in a wide variety of studies. Ion channels altered by ethanol via PKC include the metabotropic glutamate receptor, the BK-channel and the GABA chloride channel [26]. Thus, in our current study and in the literature, there is a clear link between ethanol, PKC and sodium-nutrient co-transporters. However, further studies are required to fully elucidate the mechanism of action between ethanol, B0AT1 and PKC.

## 5. Conclusions

In all, the sodium-dependent glutamine co-transporter B0AT1 was significantly inhibited by moderate ethanol in intestinal epithelial cells ex vivo and in vitro. The mechanism of inhibition was due to a decrease in the number of BBM B0AT1 co-transporters as well as a diminished sodium gradient set by the Na-K-ATPase. Furthermore, the PKC pathway likely mediates this inhibition of B0AT1 by moderate ethanol, which provides evidence that even moderate doses of ethanol affect important nutrient uptake pathways in intestinal epithelial cells and thus may contribute to alcohol-mediated malnutrition in alcoholics. Clearly, this has implications on the future treatment of malnutrition in chronic alcoholics.

## Figures and Tables

**Table 1:** The effect of moderate ethanol on the kinetic parameters of sodium-dependent glutamine co-transport in SD rats.

	*V_max_* (nmol/mg Protein·6 sec)	*K_m_* (mM)
Control	3.2 ± 0.1	26 ± 1.4
Ethanol	2.6 ± 0.1 *	26 ± 2.2

Sodium-dependent glutamine uptake was conducted using varying concentrations of glutamine with or without a sodium-free condition in BBMVs isolated from rats treated with moderate ethanol or water (control) for one-hour. Uptakes were conducted over six seconds. The maximal rate of uptake (*V_max_*) of sodium-dependent glutamine co-transport was significantly reduced when exposed to one-hour of ethanol (* *p* < 0.01, *n* = 4). There was no significant change in *K_m_* (*p* > 0.05, *n* = 4).

**Table 2:** The effect of moderate ethanol on the kinetic parameters of sodium-dependent glutamine co-transport in IEC-18 cells.

	*V_max_* (nmol/mg Protein·30 sec)	*K_m_* (mM)
Control	1.5 ± 0.03	0.1 ± 0.01
Ethanol	1.3 ± 0.03 *	0.1 ± 0.01

Sodium-dependent glutamine uptake was conducted using varying concentrations of glutamine with and without a sodium-free condition in the presence and absence of moderate ethanol exposure for one-hour. Uptakes were conducted over thirty seconds. The maximal rate of uptake (*V_max_*) of sodium-dependent glutamine co-transport was significantly reduced when exposed to one-hour of ethanol (* *p* < 0.01, *n* = 4). There was no significant change in *K_m_* (*p* > 0.05, *n* = 4).

## Appendix A. Supplemental Materials and Methods

### Appendix A.1. Acetaldehyde Measurement

Acetaldehyde was measured following one hour of ethanol exposure in IEC-18 cells using a commercial kit (Bioassays Systems).

### Appendix A.2. PKCα Western Blots

Western blots were conducted as described above. The membranes were incubated with rabbit monoclonal primary antibodies against PKCα (Abcam 4124) at 1:1000 dilution at 4°C overnight. Membranes were washed and incubated with horseradish peroxidase-conjugated anti-rabbit secondary antibodies at 1:1000 dilution at room temperature for one hour, exposed and imaged as previously described.

### Appendix A.3. Trypan Blue Exclusions

Control and ethanol-treated IEC-18 cells were washed with pre-warmed 1X PBS. Then, the cells were incubated in 0.05% trypsin in PBS for 5 min at 37°C. The resulting cell suspensions were mixed 1:1 with trypan blue solution (MilliporeSigma) and the stained cells were immediately counted with a hemocytometer.
